# Cost Analysis of Low-Volume Versus Standard-Volume Ultrasound-Guided Interscalene Brachial Plexus Block in Arthroscopic Shoulder Surgery

**DOI:** 10.7759/cureus.38534

**Published:** 2023-05-04

**Authors:** Pablo Oliver-Fornies, Alba Sánchez-Viñas, Roberto Gomez Gomez, Juan Pablo Ortega Lahuerta, Diego Loscos-Lopez, Cristian Aragon-Benedi, Ece Yamak Altinpulluk, Mario Fajardo Perez, Ignacio Aznar-Lou

**Affiliations:** 1 Department of Anesthesiology, Critical Care and Pain Medicine, Hospital Universitario de Mostoles, Madrid, ESP; 2 Health Technology Assessment in Primary Care and Mental Health (PRISMA) Research Group, Institut de Recerca Sant Joan de Deu, Barcelona, ESP; 3 Department of Anesthesiology, Critical Care and Pain Medicine, Miguel Servet University Hospital, Zaragoza, ESP; 4 Department of Anesthesiology, Critical Care and Pain Medicine, Lozano Blesa University Clinical Hospital, Zaragoza, ESP; 5 Medicine, UltraDissection, Madrid, ESP; 6 Pain Medicine, Morphological Madrid Research Center (MoMaRC), Madrid, ESP; 7 Anesthesiology Research Office, Ataturk University Medical School, Erzurum, TUR; 8 Outcomes Research Consortium, Cleveland Clinic Foundation, Cleveland, USA

**Keywords:** randomized clinical trial, limited societal perspective, interscalene brachial plexus block, economic health evaluation, diaphragmatic paralysis, costs and cost analysis

## Abstract

Background

Economic evaluation has become an essential decision-making tool for health systems worldwide. This study was aimed at estimating the difference in the use of healthcare resources, days on sick leave, and costs between patients undergoing a standard-volume versus a low-volume ultrasound-guided interscalene brachial plexus block.

Methods

This is a post-hoc cost analysis of a double-blind, randomized, and controlled clinical trial. Forty-eight patients undergoing ultrasound-guided interscalene block received either 10 ml or 20 ml of levobupivacaine 0.25%. Analyses involved the public healthcare payer perspective (including visits to general practitioners, nursing staff, physiotherapy facilities, hospital admissions, outpatient diagnostic tests, etc.) and the limited societal perspective, including productivity losses (days on sick leave). Measurements were made at one-month and one-year follow-ups post-intervention. Differences in costs were estimated using two-part models adjusted by the costs incurred in the previous year.

Results

Subjects in the 10 ml group made greater use of general practitioner visits (mean difference [95% CI]: 3.35 [0.219 to 6.49]; p=0.036) and diagnostic tests (2.43 [0.601 to 4.26]; p=0.009), but less use of physical therapy (−12.9 [−21.7 to −4.06]; p=0.004). Mean (SD) cost differences from the public healthcare payer’s perspective were 1,461.34 $ (1,541.62) and 1,024.08$ (943.83) for the 10 ml and 20 ml groups, respectively (p=0.293). From the limited societal perspective, the differences were as follows: 7,036.53$ (8,077.58) and 8,666.56$ (9,841.10), respectively (p=0.937). While there were no differences in the above parameters at the one-month follow-up.

Conclusion

The volume reduction proposed following interscalene block resulted in meaningful, albeit not statistically significant, clinical benefits and lower costs from a limited societal perspective for shoulder surgery. Thus, healthcare use and days on sick leave are variables to be taken into consideration when calculating the economic impact of surgical procedures.

## Introduction

Nowadays, the challenge faced by healthcare systems worldwide is to provide enhanced medical care in an efficient way. Health economic evaluation in the field of anesthesia is increasingly used for decision-making and has become a relevant component of healthcare system programs worldwide [1]. However, little is known about the economic impact of regional anesthesia [2] in general and, even less, about that of the brachial plexus block in particular [1,3-6]. As far as the latter is concerned, the economic outcomes reported in the literature are either rather heterogeneous [1,5,6] or evaluate obsolete regional anesthesia techniques [4]. In the only randomized clinical trial assessing the economics of ultrasound-guided interscalene block in arthroscopic shoulder surgery published to date, Gonano et al. concluded that interscalene block, as solo anesthesia, is a cost-effective technique as compared with general anesthesia [3].

Economic studies may evaluate interventions from different perspectives, such as the patients’, an institution’s, the healthcare payors’, the healthcare system’s, public health’s, or that of society in general [7]. The healthcare payor perspective, which is the main perspective used in anesthesiology studies, only considers direct medical care costs, mainly hospital costs.

The current body of literature on this topic is limited, with few available studies focusing on short-term clinical outcomes. They usually take into consideration only the costs associated with the immediate perioperative period, such as the anesthesiologist's salary [6] or the price of anesthetic drugs [1] and equipment [8], instead of considering long-term economic implications. On the other hand, the societal perspective considers broader costs to society, including both direct and indirect costs such as days of sick leave, as a proxy for productivity losses, which could represent up to 30% of costs [7]. To afford these expenses, most industrialized countries have a national policy mandating paid sick leave for workers [9]. The impact of productivity losses is alarming and remains one of the most urgent public health issues. To date, no study in the field of anesthesia has either considered indirect costs or taken a societal perspective.

In our earlier work, we demonstrated that reducing from 20 to 10 ml the volume of levobupivacaine 0.25% used for an ultrasound-guided interscalene block decreases the incidence of postoperative complications such as hemidiaphragmatic paralysis while providing non-inferior analgesia [10]. This post-hoc analysis was aimed at gaining a better understanding of the economic impact of anesthesia management in the context of arthroscopic shoulder surgery. The first step toward this goal was to estimate the difference in the use of healthcare resources and days on sick leave between the patients undergoing standard-volume and low-volume ultrasound-guided interscalene blocks included in the REDOLEV trial [10]. As a second step, an estimation was made of the difference between the total costs for both groups under the public healthcare payor and limited social perspectives.

## Materials and methods

Study design

This is a post-hoc cost analysis of the REDOLEV trial, a phase III double-blind randomized prospective single-centre two-arm comparative controlled clinical trial [7,10]. The present report was drafted following the Consolidated Health Economic Evaluation Reporting Standards (CHEERS) Statement [7]. This study was approved by the Ethics Committee for Clinical Research of Aragon (reference number: EC19/093 with principal investigator Pablo Oliver-Fornies; date: December 18, 2019). The paper was registered on ClinicalTrials.gov (NCT04385966) and on the European Union Drug Regulating Authorities Clinical Trials Database (2019-003855-12).

Setting

The study was conducted from February 2020 to November 2021 at a single public orthopedic hospital in Saragossa (Spain). Saragossa is the capital city of Aragon, a Spanish autonomous region. The Spanish public healthcare system provides universal coverage to both national and foreign citizens. It is funded mainly through taxes and is free of charge at the point of use, with some exceptions, such as outpatient medication. The system is decentralized; thus, each of the 17 Spanish autonomous regions controls health planning, public health policies, and the management of health services [11].

Subjects

Inclusion criteria were as follows: patients aged 18 to 80 years old; ASA I-III; scheduled for elective in-patient arthroscopic shoulder surgery and interscalene block. Exclusion criteria were: age <18 and >80 years old; pregnancy; inability or contraindication to undergo interscalene block or spirometry; allergy to the anesthetic drugs used in the study; history of moderate or severe pulmonary disease; and chronic opioid consumption.

After confirming eligibility, written informed consent was obtained from all subjects. Randomization was accomplished using opaque sealed envelopes with computer-generated randomization allocation of eligible patients to the two study arms. Patients were randomly allocated in equal proportions to one of the two arms: the control group (n=24) received a standard-dose interscalene block (20 ml of levobupivacaine 0.25%) at the C5-C6 level in a single shot, while the intervention group (n=24) received a low anesthetic dose (10 ml of levobupivacaine 0.25%). The study was double-blinded in that trial participants, data collectors, outcome adjudicators, and data analysts were kept blinded. The only unblinded researchers were the anesthesiologists administering the anesthetics.

Data collection

Data collected included patients’ sociodemographic and clinical data, medications used, days on sick leave, and healthcare use. Sociodemographic and clinical data were obtained from the primary analysis (REDOLEV trial), the postanesthesia care unit (PACU), and the hospital ward.

Economic data were collected from electronic health records of patients enrolled in the REDOLEV trial 12 months before and 1 and 12 months after the intervention. The number of days on sick leave was also obtained from electronic medical records, as, in Spain, general practitioners are responsible for certifying sick leaves.

Outcomes

Cost analyses were conducted from the public healthcare payor’s perspective, i.e., including only direct medical costs, and from the limited societal perspective, which included direct medical costs and indirect costs [12,13]. We collected data from the following healthcare resources: onsite and home visits with the general practitioner and nursing services in the primary and secondary care settings; emergency room visits; use of physical rehabilitation and physiotherapy sessions; inpatient hospital admissions (including lengths of stay); and outpatient diagnostic tests (radiographs and blood tests). The duration of PACU and hospital stays was also recorded. Indirect costs included productivity losses (such as days on sick leave). We conducted the analysis following the intention-to-treat principle, including all patients who fulfilled the inclusion criteria.

Our co-primary outcomes were the costs associated with the use of healthcare resources and days on sick leave. Secondary outcomes included other costs, such as medication costs, etc. The healthcare resources used were converted to monetary costs by multiplying each item by its tariffs (used as unit costs), as published in Aragon’s Official Gazette [14,15]. When the gazette did not specify any charge (which was the case for radiographs and blood tests), the mean of the tariffs published in the gazettes of the remaining Spanish regions was used [16-21]. The tariffs used were those corresponding to the most recent publication. To estimate the cost from a societal perspective, the number of days on sick leave was used as a proxy for productivity losses. Sick leave was converted to monetary costs by using the minimum daily wage in Spain corresponding to 2021 [22,23]. A sensitivity analysis was undertaken, considering the mean monthly wage in Aragon for 2020 to be the unit cost for days on sick leave. Unit costs were estimated in Euros (€) and updated according to the 2022 (March) Spanish Consumer Price Index. Pricing information regarding the medication used was obtained from the hospital’s pharmacy (Table 1).

**Table 1:** Unit costs for health services and drugs use in the cost analysis. *Costs were collected in Euros (€) and they were translated to United States dollars (USD or $) using InforEuro which is based on an official change published by the European Central Bank on March 2022 (1€ = 1.1216 USD) [24]. **The secondary analysis with a societal perspective accounts for the total healthcare costs included in the healthcare perspective and the cost of sick leaves, using the average daily wage in Aragon.

Health services	Unit costs (USD: $)*	Source (reference)
General practitioner		[14]
Primary care centre		
First visit	89.16	
Follow-up visit	44.56	
Home visit		
First visit	106.98	
Follow-up visit	53.51	
Nurse		[14]
Primary care centre	41.13	
Radiography	17.83	[16-21]
Blood test	72.30	[16-21]
Secondary care visit**	36.41	[15]
Physical rehabilitation	6.92	[15]
Physiotherapy	6.92	[15]
Inpatient hospital admissions: $/day	135.92	[15]
Hospital emergency room	48.54	[15]
Sick leave (min Spain): $/day	37.17	[22]
Sick leave (mean Aragón): $/day	78.76	[23]
Drugs		
Levobupivacaine 0.25% 10 ml	0.66	
Morphine 10 mg	0.21	

Costs were calculated based on the assumption that the drug left over in a single-use ampoule had to be discarded [3]. Direct costs related to the operating room, staff salaries, and drugs administered in the perioperative period, except for the drug under analysis and the postoperative analgesics, were not included because both groups received the same perioperative treatment as per the study protocol. The unit cost of the health resources used was limited to the study setting, the Aragon Healthcare Service (SALUD), and the Spanish National Health System. Costs were translated to United States dollars (USD or $) using InforEuro, which is based on the official change published by the European Central Bank on March 2022 (1€ = 1.1216$) [24].

Statistical analysis

The healthcare use and cost differences between groups were assessed one month and one year after the intervention. Due to the variable distribution and the high proportion of zeros in the sample, mean differences in healthcare use and days on sick leave between groups were estimated using two-part models. A preliminary logistic regression was applied to obtain the probability of using a given healthcare service. These analyses were followed by generalized linear models, with the Poisson distribution performed conditional on the positive outcome of the first part. The models were controlled for baseline healthcare resource use (those incurred in the 12 months preceding the intervention).

The cost analysis was performed using generalized linear models with gamma distributions, a logistic link, and costs as the dependent variable. The models were controlled for baseline costs (those incurred in the 12 months preceding the intervention). Differences in operating room and length of stay in the PACU between the intervention and control groups were assessed through a t-test. The method followed to estimate the sample size (a power of 90% and a dropout rate of 10%) was specified in a previous article [10]. For all analyses, a statistically significant result was assumed if p<0.05. Analyses were performed using the Stata MP software package (version 17.0) (StataCorp LLC, Texas, USA).

## Results

**Figure 1:**
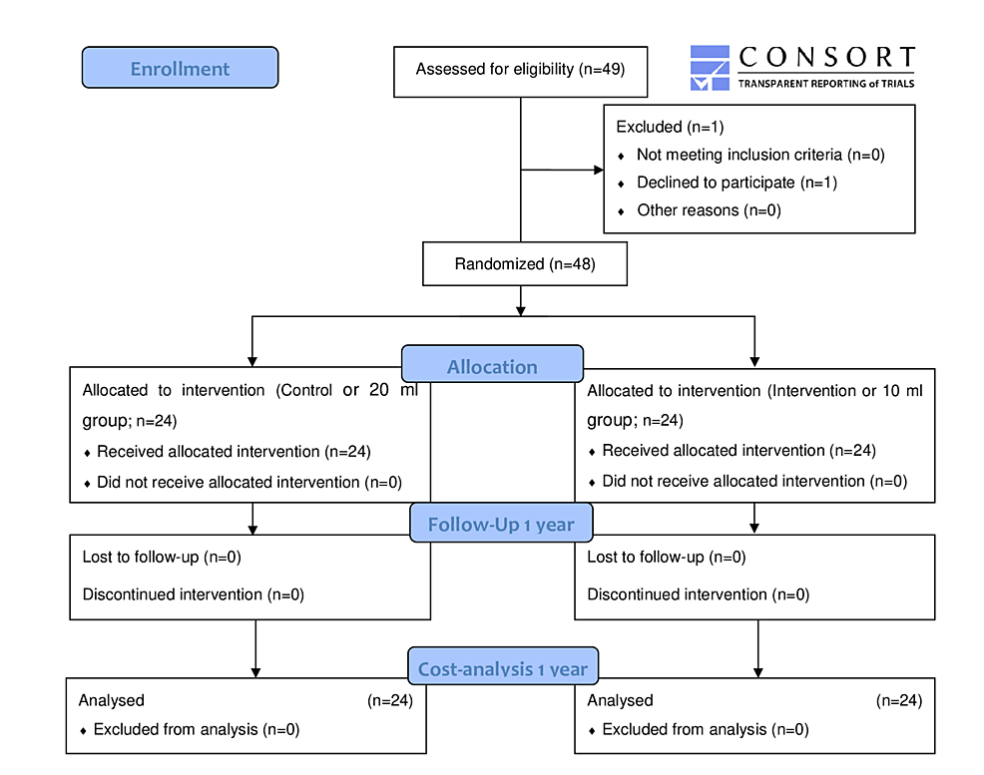
CONSORT diagram of patient recruitment.

A total of 49 subjects were screened for recruitment (first recruitment: February 11, 2020). During the recruitment phase, one patient declined to participate. Therefore, written informed consent was obtained from 48 subjects, who were randomly allocated in equal numbers to each group and followed up until one year after the procedure (Figure 1).

Participant characteristics were balanced between intervention groups. Surgery mostly consisted of rotator cuff repairs. All participants recruited had a successful interscalene block and experienced no short-term complications following the surgical procedure. No delayed hospital discharge occurred in either group. All primary and secondary clinical outcomes were duly assessed [10]. During follow-up, no difference was observed between the groups in the influence of the COVID-19 pandemic (p = 0.296). The median (IQR) time from surgery to the last orthopedic consultation was 100 (142) days for the 10 ml group and 138 (115) days for the 20 ml group (p = 0.775). The two cohorts were comparable at baseline [10]. No subgroup analysis was therefore needed.

Healthcare use and days on sick leave

The use of healthcare services and days on sick leave are summarized in Table 2.

**Table 2:** Impact of intervention (reduction of local anesthetic volume after ultrasound-guided interscalene block) on healthcare use and days on sick leave. Use of services data is expressed as mean (SD: standard deviation). *Diagnostic tests include radiography and blood test. (-) Analysis is not available due to a lack of data.

Time point	Control or 20 ml group (n=24)	Intervention or 10 ml group (n=24)	Use of service difference	p-value
One-month follow-up	Mean (SD)		Mean difference (95% CI)	
General practitioner	2.25 (2.15)	2.17 (3.24)	−0.224 (−1.41 to 0.961)	0.711
Nurse	1.08 (1.05)	1.79 (1.93)	0.303 (−0.608 to 1.21)	0.514
Diagnostic tests*	0.292 (0.690)	0.083 (0.282)	−0.159 (−0.578 to 0.259)	0.455
Secondary care	2.08 (1.18)	2 (0.885)	−0.079 (−0.950 to 0.791)	0.858
Rehabilitation medicine	-	-	-	-
Physical therapy	-	-	-	-
Sick leave (days)	18.13 (14.66)	15.46 (14.95)	−1.95 (−9.64 to 5.75)	0.620
One-year follow-up				
General practitioner	10.75 (11.63)	17.75 (11.63)	3.35 (0.219 to 6.49)	0.036
Nurse	0.458 (0.833)	1.38 (2.43)	0.422 (−0.476 to 1.32)	0.357
Diagnostic tests*	2.96 (6.02)	4.83 (5.57)	2.43 (0.601 to 4.26)	0.009
Secondary care	4.88 (5.82)	5.21 (5.48)	0.511 (−0.954 to 0.747)	0.494
Rehabilitation medicine	3.21 (3.46)	2.54 (2.67)	−0.463 (−1.88 to 0.953)	0.522
Physical therapy	19.86 (25.84)	7.58 (13.17)	−12.9 (−21.7 to −4.06)	0.004
Sick leave (days)	97.04 (117.8)	70.79 (97.57)	−21.2 (−60.7 to 18.4)	0.295

After a one-month follow-up, no statistically significant differences were observed between groups. Among those patients receiving 10 ml, the use of a general practitioner was higher at one-year follow-up, with a mean difference (95% CI) of 3.35 (0.219 to 6.49) (p = 0.036). The use of diagnostic tests was also higher (2.43 [0.601 to 4.26]; p = 0.009). The 10 ml group made less use of physical therapy (−12.9 [−21.7 to −4.06]; p = 0.004).

Although not statistically significant, the 10 ml group spent fewer days on sick leave at one month (mean difference [95% CI]; −1.95 [−9.64 to 5.75]) and 1-year time horizon (−21.2 [−60.7 to 18.4]). Indeed, more participants in the 20 ml group were absent from work for more than 30 days than in the 10 ml group (14/24 [58.3%] vs. 10/24 [41.7%]).

Differences in costs

Table 3 reports the cost analysis of both study groups during the follow-up period. Data from unadjusted analyses are available from the authors on request.

**Table 3:** Mean cost and mean cost difference between low-volume and standard-volume local anesthetic after ultrasound-guided interscalene block. A cost analysis from Healthcare and Societal perspectives. Cost data are expressed as mean (SD: standard deviation). Cost differences are expressed as mean (95% confidence interval). *P‐value <0.05 was considered statistically significant. *The healthcare perspective includes visits to primary care clinician (PCC/home), nurse visits, radiographies, blood tests, inpatient hospital admissions (PCC/home), secondary care visits, physiotherapy and rehabilitation sessions and Hospital ER. **The main analysis with a societal perspective accounts for the total healthcare costs included in the healthcare perspective and the cost of sick leaves, using the minimum daily wage in Spain. ^†^Costs were collected in euros (€) and they were translated to United States dollars (USD or $) using InforEuro which is based on official change published by the European Central Bank on March 2022 (1€ = 1.1216 USD) [24].

Healthcare system perspective*			Societal perspective**†			
			Minimum wage		Mean wage	
Healthcare use during the previous year						
Group	Costs ($)	p-value	Costs ($)	p-value	Costs ($)	p-value
Control or 20 ml group	947.34 (867.11)	-	2,777.69 (4005.78)	-	4,826.01 (7794.12)	-
Intervention or 10 ml group	1,137.16 (940.16)	-	2,631.48 (4298.27)	-	4,303.76 (8255.40)	-
Healthcare use at 1 month follow-up						
Group	Costs ($)	p-value	Costs ($)	p-value	Costs ($)	p-value
Control or 20 ml group	277.12 (145.26)	-	950.72 (601.01)	-	1,704.55 (1202.27)	-
Intervention or 10 ml group	292.03 (203.75)	-	866.53 (602.95)	-	1,509.44 (1206.49)	-
Mean Cost difference (95% CI)	0.16 (−100.76; 101.07)	0.998	−64.78 (−421.59; 292.01)	0.722	−132.05 (−853.65; 589.64)	0.720
Healthcare use at 1 year follow-up						
Group	Costs ($)	p-value	Costs ($)	p-value	Costs ($)	p-value
Control or 20 ml group	1,024.08 (943.83)	-	4,630.56 (4,971.93)	-	8,666.56 (9,841.10)	-
Intervention or 10 ml group	1,461.34 (1,541.62)	-	4,092.28 (4,163.02)	-	7,036.53 (8,077.58)	-
Mean cost difference (95% CI)	331.04 (-285.50; 947.58)	0.293	389.60 (−2,529.34; 3,308.34)	0.794	231.37 (−5,540.26; 6,003.01)	0.937

After one-year follow-up, the mean (SD) total cost from the public healthcare payor’s perspective was 1,461.34$ (1,302.91€) (SD: 1,541.62$ [1,374.48€]) and 1,024.08$ (913.05€) (SD: 943.83$ [841.50€]) in the 10 ml and 20 ml groups, respectively. The mean cost difference was not statistically significant (p = 0.293). From the limited societal perspective, the mean total cost for the 10 ml group was not statistically significant but lower than that for the 20 ml group (7,036.53$ [6,273.65€] [SD: 8,077.58$ {7,201.84€}] versus 8,666.56$ [7,726.96€] [SD: 9,841.10$ {8,774.16€}]; p = 0.937), using the average daily wage.

Per-patient medication costs (levobupivacaine 0.25%) per-patient were 0.66$ (0.59€) and 1.32 $ (1.18€) for the 10 ml and 20 ml groups, respectively. Regarding postoperative intravenous morphine consumption, estimated per-patient costs were equal for both groups (0.21 $ [0.19€] [SD: 0.21 $ {0.19€}]). As aforementioned, apart from these differences in costs, there was no direct cost difference associated with the anesthesia employed. Median (IQR) length of stay in the PACU (60 [37.5] versus 80 [52.5] minutes; p = 0.412) and in the hospital ward (28.5 [16] versus 30 [16] hours; p = 0.604) were not significantly different between groups.

## Discussion

This study suggests that there is a difference between low- and standard-volume ultrasound-guided interscalene brachial plexus blocks in terms of healthcare use. When considering only the public healthcare payor’s perspective, the total cost for the 10-ml group was slightly higher than that for the 20-ml group at one-year follow-up. However, this result is reversed from a limited societal perspective (i.e., by adding indirect costs). The 20-ml group seems to have incurred higher expenditure costs at one-year follow-up than the 10-ml group. These differences were nonetheless not statistically significant.

Shoulder arthroscopy is associated with significant postoperative pain and can affect early postoperative rehabilitation [25]. First, it could be hypothesized that lower-volume regional blocks may be associated with less pain relief and, consequently, significantly higher procedure-related costs. However, this was not the case in our study [10]. Perioperative anesthesia costs account for about 6% of total costs [26]. Our analysis showed a minimum economic impact of postoperative analgesia (less than $1). Our findings seem to confirm that direct medication costs are sometimes negligible in the field of anesthesia [3]. Additionally, PACU and in-ward lengths of stay were equal for both groups. Even then, it is unclear whether a reduced stay results in any cost reduction [3].

Second, early rehabilitation and functional recovery are now widely recognized as the primary goals after shoulder surgery [27]. However, the long-term benefits of regional anesthesia are debatable [27]. Gurger et al. found that interscalene block provided an improvement in shoulder function at week 6 after surgery [28]. However, we were unable to detect any overall cost difference after one month. This may be explained by the fact that our evaluation was underpowered. Other studies found no benefits for up to six months [29]. We found that subjects in the 10 ml group needed fewer visits to rehabilitation and physical therapy at one-year follow-up. As previously shown, higher volume blocks can be associated with a higher rate of complications [10]. To explain the higher expenditure costs found in the 20-ml group, one plausible reason is that functional recovery influences the use of healthcare services. Nonetheless, these can only be speculations, as the study was limited to economic rather than functional outcomes. It is therefore difficult to explain a potential interscalene blockade-related interference with long-term rehabilitation.

Beyond the clinical benefits shown in trials, medical improvements are always associated with societal implications. Nowadays, productivity losses represent one of the most important costs in the perioperative process [9]. This impact could reach up to 30% of costs [30]. Using the limited societal analysis, we found a positive economic impact in the 10 ml group after a one-year follow-up. This difference was mainly due to a lower number of days on sick leave. We have raised many hypotheses to correlate these findings with clinical outcomes. However, neither differences in pain relief, the rate of complications, nor functional recovery seem to be potential causes. Long-term anesthesia results are sometimes difficult to understand, and the present cost analysis is similarly hard to interpret.

Although controversy still persists as to what kind of block is recommended for shoulder surgery, in earlier work we demonstrated that the low-volume technique is effective. Thanks to this analysis, we can now affirm that it can be implemented at no extra cost. However, the long-term functional benefits of diaphragm-sparing regional strategies are yet to be explained.

The primary limitation of this study is its post-hoc nature. Indeed, the sample size was inevitably small because it was powered to measure a clinical effect rather than an economic impact as a primary endpoint. Therefore, we should remain cautious when interpreting the findings obtained. Second, this cost analysis was performed with a particular setting in mind, i.e., that of a predominantly government-funded healthcare system offering universal coverage, with some relevant costs, such as those involved in the provision of formal or informal care, not being considered. This can limit the generalizability of the study's findings to other settings. Third, shoulder function was not assessed during follow-up using scores or questionnaires [28].

Although the present study is a mere description of the costs incurred after regional anesthesia, this is the first analysis to assess a regional anesthesia technique from both the public payor’s and limited societal perspectives.

A well-designed health economics analysis evidently requires a broader dataset, encompassing survival and quality-of-life measures and quantification of post-discharge use of resources over an extended follow-up period. In this regard, the use of real-world data from electronic medical records is a very promising opportunity to evaluate the economics of anesthesiology interventions. We believe that this study conveys valuable information that can guide future research.

## Conclusions

In short, the present cost analysis shows that healthcare use and days on sick leave are variables to be taken into consideration when calculating the economic impact of surgical and anesthetic procedures. Although this is a small-scale, single-center study, the volume reduction obtained resulted in meaningful, albeit not statistically significant, clinical benefits and lower costs from a limited societal perspective. This means that the direct and indirect costs incurred during the postoperative period could be reduced by implementing a strategy that, additionally, improves patient safety.
